# Pancytopenia in a 70-Year-Old African-American Male: An Unusual Presentation of a Rare Disease

**DOI:** 10.1155/2014/354810

**Published:** 2014-02-16

**Authors:** Aibek E. Mirrakhimov, Alaa M. Ali, Aram Barbaryan, Nwabundo Anusim, Raya Saba, Shawn G. Kwatra, Nasir Hussain, Teresita Zdunek, Alan D. Gilman

**Affiliations:** ^1^Department of Internal Medicine, Saint Joseph Hospital, 2900 North Lake Shore Drive, Chicago, IL 60657, USA; ^2^Department of Dermatology, Johns Hopkins University School of Medicine, Baltimore, MD 21287, USA; ^3^Department of Pathology, Saint Joseph Hospital, 2900 North Lake Shore Drive, Chicago, IL 60657, USA; ^4^Division of Hematology and Oncology, Saint Joseph Hospital, 2900 North Lake Shore Drive, Chicago, IL 60657, USA

## Abstract

Hairy cell leukemia is a rare lymphoid neoplasm arising from mature B-lymphocytes. Clinically, the disease presents with splenomegaly and abdominal discomfort, frequent infections, fatigue and bleeding because of related cytopenias. Bone marrow biopsy is essential for diagnosis. Below we describe a case of a 70-year-old African-American male who presented to our hematology clinic complaining of fatigue. Clinical exam and computed tomography imaging did not reveal splenic enlargement. Blood work-up revealed pancytopenia and bone marrow was diagnostic for hairy cell leukemia.The patient was started on cladribine, with gradual improvement of his symptoms and blood count abnormalities. Therefore, it is essential to keep hairy cell leukemia in the differential of pancytopenia even in the absence of a splenomegaly.

## 1. Introduction

Hairy cell leukemia (HCL) is a rare lymphoproliferative disorder originating from B-lymphocytes and accounts for less than 1% of lymphoid malignancies in The United States [1]. The disease's name is derived from characteristic hairy cytoplasmic projections of malignant B-lymphocytes. It is relevant to mention that the disease is more common among people of Caucasian and Ashkenazi Jewish-descent [2].

The pathogenesis of HCL is not completely understood. However, several pathologic factors play a role in the development of this disease. First, mutations in BRAF proto-oncogene were found to be implicated in the pathogenesis of HCL [3]. Overactivation of BRAF pathway leads to uncontrolled cellular proliferation. In addition to that, HCL cells produce various cytokines such as basic fibroblast growth factor, transforming growth factor, and tumor necrosis factor alpha which lead to fibrosis and suppression of the bone marrow with resultant pancytopenia [4]. Some patients with HCL may have abnormalities of fifth chromosome [4].

It is interesting to note that the malignant cell of HCL is a mature B-lymphocyte at a late stage of development (up to preplasma cell) [4]. These cells express cluster of differentiation (CD) antigens characteristic of a mature B-lymphocyte such as CD 19, CD 20, and CD 22 as well as CD 11c, CD 25, and CD 103 [5]. HCL cells do not possess CD antigens of B-lymphocytes at earlier stages of development such as CD 10 and CD 21 and plasma cells such as CD 5 and CD 23 [6, 7]. Therefore, it is believed that hairy cells are clonal Blymphocytes arrested at a late stage of development [2].

Clinical presentation of HCL is nonspecific and is related to cytopenias [6]. Affected patients may complain of fatigue, easy and prolonged bleeding, easy bruises, frequent infections, and abdominal discomfort. The vast majority of patients with HCL have splenomegaly (prevalence ranges from 66% to 100%) [8–11]. Furthermore, splenomegaly tends to be massive in patients with HCL [12]. However, hepatomegaly and lymphadenopathy are rarely encountered in patients with HCL [8]. General laboratory work-up will reveal anemia, thrombocytopenia, neutropenia, and monocytopenia in the vast majority of patients. On a rare occasion, patients may present with leukocytosis [8]. Peripheral smear will show hairy cytoplasmic projections in at least 90% of the cases [13]. Bone marrow (BM) examination is necessary to establish the diagnosis of HCL. BM aspiration often is “dry” because of fibrosis and BM biopsy shows hypercellular marrow in most of the cases [14].

The decision to treat should be based on several factors such as symptomatic cytopenias manifested as frequent infections, fatigue, easy bleeding, splenomegaly and abdominal discomfort, fevers, and night sweats [6]. In asymptomatic cases, it may be reasonable to watch the patient closely for disease progression since efficacy of early treatment in such patients is controversial. Several treatment options are available such as chemotherapy and splenectomy as a last resort. Chemotherapy choices consist of cladribine monotherapy, pentostatin monotherapy, or interferon alpha monotherapy. For a greater coverage of the topic on HCL treatment, the reader is referred to a well written article [6].

Below we present a case of 70-year-old African-American male who was referred to our clinic because of pancytopenia of 3-month duration. We will review other cases of HCL in which splenomegaly was not reported.

## 2. Case Presentation

A 70-year-old never-smoker African-American male with past medical history of type 2 diabetes mellitus (DM), hypertension (HTN), and hyperlipidemia (HLD) was referred to our clinic because of a 3-month history of pancytopenia which was accidentally detected during his regular visit to a primary care physician (PCP). The patient was drinking no more than 3 beers a week and never used any recreational drugs. The previous blood work-up was done approximately 1 year ago at his PCP office. The patient's vaccination history was up to date.

Familial medical history was positive for type 2 DM in his father and was negative for any blood or oncological disorder. The patient was using 500 mg of metformin for type 2 DM, lovastatin 10 mg for HLD, and 10 mg of lisinopril for HTN. There were no recent changes in medications (no changes in 2 years) and no use of over-the-counter or herbal medications. HTN, HLD, and type 2 DM were well controlled with the above medications. The patient's last colonoscopy was done 1 year ago and was unremarkable. The patient denied any prior exposure to radiation, chemotherapy, blood transfusion, or BM analysis in the past.

On a review of systems the patient denied having chest pain, shortness of breath, cough, fever, chills, night sweats, weight change, abdominal discomfort, early satiety, nausea, vomiting, diarrhea, constipation, rash, easy bruising or bleeding, bloody stools, frequent infections, and fatigue. Upon checking his vital signs blood pressure was 122/68, heart rate was 74, respiratory rate was 12, temperature was 97.6 F (36.4°C), and oxygen saturation 97% in room air. The patient's body mass index was 26.1 kg/m^2^. Physical examination was unremarkable and negative for lymphadenopathy and organomegaly.

Complete blood count (CBC) analysis at the clinic showed white blood cell (WBC) count of 2.200 cells/mL (normal range: 4.000–11.000), platelets of 99.000 cells/mL (normal range: 150.000–450.000), and hemoglobin (Hb) of 12.1 g/dL (normal range: 13.0–17.0). Manual differentiation of CBC showed mild neutropenia of 700 cells/mL (normal range: 1.500–8.000) and absolute monocytopenia. Review of peripheral smear was noncontributory and negative for schistocytes or hairy cells.

Mean corpuscular volume (MCV) of red blood cells (RBC) was 103.1 (normal range: 80–100). Comprehensive metabolic panel (CMP) was unremarkable with normal liver function tests including bilirubin. Further blood work-up included serum vitamin B12, RBC folate, anti-nuclear antibody, erythrocyte sedimentation rate (ESR), human immunodeficiency virus serology, serologies for hepatitis viruses, cytomegalovirus serology, Ebstein-Barr virus serology, uric acid, haptoglobin, lactate dehydrogenase, Coombs test, serum protein electrophoresis, and serum immunoglobulins. Other than mildly elevated ESR at 21 mm/hour (normal range: 0–12) all tests were within normal limits.

**Figure 1:**
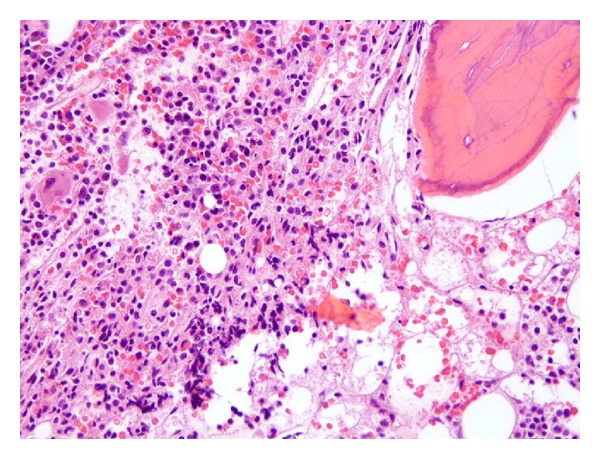
Hypercellular bone marrow.

**Figure 2:**
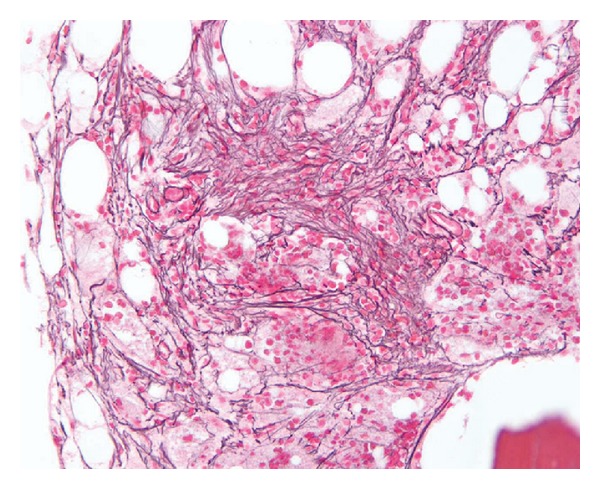
Increased reticulin deposition resulting in fibrotic bone marrow.

BM aspiration and biopsy were done for further work-up of pancytopenia. BM review showed mildly hypercellular BM (please see Figure 1) with infiltration by atypical lymphocytes and increased reticulin fibrosis (please see Figure 2). Flow cytometry showed a population of atypical lymphocytes positive for CD 19, CD 25, CD 11c, and CD 103. Fluorescence in situ hybridization (FISH) analysis showed deletion of immunoglobulin heavy chain (IGH) on the long arm of fourteenth chromosome (14q32). Polymerase chain reaction revealed BRAF V600E mutation.

**Figure 3:**
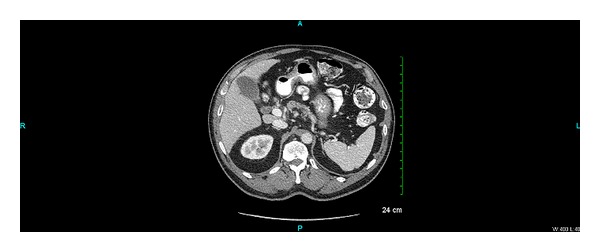
Abdominal and pelvic CT scan showing normal sized liver and spleen.

Computed tomography (CT) scan of the abdomen and pelvis was ordered to confirm the absence of organomegaly. CT scan was unremarkable and negative for any organomegaly and lymphadenopathy (please see Figure 3).

The patient tolerated chemotherapy well, with the lowest WBC of 1.1, neutrophils of 480, HB of 11, and platelets of 60. The patient did not experience any infectious complication or bleeding and was not admitted to the hospital within 5 months of treatment. The patient's blood counts improved and normalized by week 10 after treatment. The patient's counts were WBC of 4.7, HB of 13.1, and platelets of 172. The patient is doing well five months after treatment.

## 3. Discussion

Splenomegaly is a cardinal clinical feature of the vast majority of HCL and HCL is typically included in the differential diagnosis of splenic enlargement [15]. Several case reports were published presenting patients with HCL and lack of splenomegaly.

Spedini et al. presented a case of a 71-year-old male who presented with right hip pain [16]. CT scan showed lytic bone lesions in femoral neck and head. Physical examination was remarkable only for right hip tenderness. Laboratory work did not reveal cytopenias or other abnormalities. Abdominal and chest CT showed no abnormalities including splenomegaly. BM biopsy showed evidence of HCL. The patient was treated with radiation therapy and interferon alpha.

Lal et al. described a case of a 45-year-old male who presented with left hip pain [17]. The patient was found to have bone marrow occupying lesions in the left femoral neck, proximal left femur, and bilateral greater trochanters. Biopsy of the left hip revealed HCL. It is very interesting to mention that subsequent BM biopsy did not reveal any evidence of BM involvement by HCL and was generally unremarkable. The patient's blood counts were normal. Karmali et al. described another case of a 41-year-old male with isolated HCL involving bone without BM involvement and splenomegaly [18].

Rosen et al. described a case of a 54-year-old male who presented with severe back pain and numbness and weakness in his lower extremities [19]. The patient underwent a surgery for epidural lesion, which was later found to be HCL. No laboratory abnormalities and splenomegaly were found. BM biopsy was diagnostic for HCL. The patient later underwent chemotherapy with cladribine.

Osman et al. presented a case of a 39-year-old male who presented with right hip pain of 5-year duration [20]. Magnetic resonance imaging (MRI) showed an ovoid, hyperintense lesion confined to the right sacral ala with no evidence of cortical destruction. CT guided biopsy showed HCL. There was no evidence of splenomegaly on physical examination and CT scan of the abdomen and pelvis. Similarly, to the case presented by Lal et al. and Karmali et al. there was no evidence of BM involvement [17, 18].

Gray et al. reported a case of 56-year-old male who presented with chronic back pain and intermittent hip pain [21]. CT imaging revealed lytic bone lesions in vertebral bodies and a small lytic lesion within the right ilium. Laboratory work-up did not reveal cytopenia. There was no evidence of splenomegaly. Biopsy of lytic bone lesion and BM biopsy revealed HCL. The patient underwent radiation therapy, followed by cladribine and alendronate.

In conclusion, all of the above cases had evidence of bone disease and did not have any evidence of cytopenias and splenomegaly. Furthermore, three cases reported negative BM biopsy in patients with bone lesions, which were found to be HCL. In contrast, our patient had evidence of peripheral blood and BM involvement. It is necessary to keep in mind that most of the HCL cases present with splenomegaly which is often massive. Nevertheless, it is essential to remember that cases of HCL without splenomegaly do exist and astute clinical approach is recommended. HCL should be included in the differential diagnoses of pancytopenia with or without splenomegaly which is highlighted by this case. Isolated bone lesions should be biopsied and treated accordingly to the pathological diagnosis.
